# Endoglin pathway genetic variation in preeclampsia: A validation study in Norwegian and Latina cohorts^☆^

**DOI:** 10.1016/j.preghy.2017.10.005

**Published:** 2018-04

**Authors:** Mandy J. Schmella, James M. Roberts, Yvette P. Conley, Dianxu Ren, Gro L. Storvold, Sue A. Ingles, Melissa L. Wilson, Anne Catherine Staff, Carl A. Hubel

**Affiliations:** a Department of Health Promotion and Development (School of Nursing), University of Pittsburgh, Pittsburgh, PA, USA; b Department of Obstetrics, Gynecology, & Reproductive Sciences (School of Medicine), University of Pittsburgh, PA, USA; c Magee-Womens Research Institute, Pittsburgh, PA, USA; d Department of Epidemiology (Graduate School of Public Health), University of Pittsburgh, Pittsburgh, PA, USA; e Clinical and Translational Science Institute, University of Pittsburgh, Pittsburgh, PA, USA; f Department of Human Genetics (Graduate School of Public Health), University of Pittsburgh, Pittsburgh, PA, USA; g Department of Health and Community Systems (School of Nursing), University of Pittsburgh, Pittsburgh, PA, USA; h Faculty of Medicine, University of Oslo, Oslo, Norway; i Division of Obstetrics and Gynaecology, Oslo University Hospital, Oslo, Norway; j Department of Obstetrics and Gynecology (Keck School of Medicine), University of Southern California, Los Angeles, CA, USA; k Department of Preventive Medicine (Keck School of Medicine), University of Southern California, Los Angeles, CA, USA; l Department of Environmental and Occupational Health (Graduate School of Public Health), University of Pittsburgh, Pittsburgh, PA, USA; m Global Pregnancy Collaboration, USA

**Keywords:** Preeclampsia, Genetic association, Candidate gene, Endoglin, Pregnancy

## Abstract

*Objective:* The purpose of this study was to validate our previous genetic association findings related to the endoglin (*ENG*) pathway from an American Caucasian preeclampsia cohort in independent preeclampsia cohorts. We also sought to explore the *ENG* pathway for new genetic associations in these independent cohorts. *Study design:* We used a tagging single nucleotide (tSNP) approach to assess genetic variability across five *ENG* pathway genes (*ENG*, *TGFβ1*, *TGFβR1*, *ALK1*, and *TGFβR2*) in a Caucasian cohort from Norway (n = 77 preeclampsia cases & n = 63 normotensive controls) and a White Hispanic cohort from Southern California (n = 69 preeclampsia cases & n = 106 normotensive controls). *Main outcome measures:* Univariate analyses (Chi Square, Fisher’s Exact) and multivariate logistic regression were conducted to evaluate the association between tSNP genotype distributions and pregnancy outcome in each cohort. Logistic regression models were adjusted for maternal age at delivery, infant sex, parity, smoking during pregnancy, and pre-pregnancy BMI. *Results:* Although we were unable to replicate our previous SNP-specific findings (*ENG* rs11792480, rs10121110; *TGFβR2* rs6550005; p’s > 0.05), we found that genetic variation in *TGFβR1[ALK5]* (rs6478974) and *TGFβR2* (rs11129420, rs6802220, rs1155708, rs3773640, rs3773663) was significantly associated with preeclampsia in the Norwegian cohort and genetic variation in *ALK1* (rs706819) and *TGFβR2* (rs9843942) was significantly associated with preeclampsia in the Latina cohort. *Conclusion:* Overall, our results provide further support for the involvement and investigation of the endoglin pathway in preeclampsia.

## Highlights

• The endoglin pathway’s role in preeclampsia has not been fully elucidated.
• Genetic variation in endoglin pathway genes is associated with preeclampsia.
• Endoglin pathway genetic variation in preeclampsia may vary by ancestry.

Characterized as one of the “great obstetrical syndromes,” [1] preeclampsia is a heterogeneous, multi-system hypertensive disorder that affects 3–5% of pregnancies [2], [3], [4] and significantly contributes to maternal and infant morbidity and mortality worldwide [5]. Preeclampsia is diagnosed when a woman develops new-onset gestational hypertension and proteinuria, or multi-systemic signs in the absence of proteinuria, after 20 weeks’ gestation. However, the underlying pathophysiologic mechanisms that lead to the development of preeclampsia and subsequent maternal endothelial dysfunction have not been fully elucidated [5]. An imbalance of angiogenesis-related factors, including endoglin/soluble endoglin, represents one potential mechanism that has garnered a great deal of attention over the last decade [6], [7].

Endoglin (CD105; ENG) is a membrane-bound co-receptor of transforming growth factor beta (TGFβ) that regulates vascular tone [8], [9], is essential for normal angiogenesis and vascular development [10], and negatively regulates trophoblast differentiation towards the invasive phenotype [11], [12]. Throughout pregnancy, *ENG* mRNA expression is elevated in the placenta and the maternal blood (cellular and cell-free components) in women who develop preeclampsia [13], [14], [15], [16], [17], [18], [19]. A preliminary study has also demonstrated that *ENG* mRNA expression is significantly elevated in microvascular endothelial cells isolated from adipose tissue obtained at cesarean section in women with preeclampsia [20]. Moreover, soluble endoglin (sENG) protein is significantly elevated in the circulation of women with preeclampsia weeks to months before clinically overt disease [21], [22]. Soluble endoglin, which is generated by the cleavage of the *trans*-membrane ENG receptor, is postulated to enter the maternal circulation from the placenta where it sequesters transforming growth factor beta (TGFβ) and inhibits TGFβ-mediated cell signaling and endothelial function [19], [23].

These elevations in endoglin mRNA expression and circulating endoglin protein levels in women with preeclampsia, assumed to be due to the increased contribution from a dysfunctional placenta, prompted us to investigate the role that maternal genetic variation in *ENG* and other members of the TGFβ1 signaling system may play in the development of preeclampsia. Using a case-control genetic association study design and a tagging single nucleotide polymorphism (tSNP) approach, we previously found that genetic variation in *ENG* (rs11792489, rs10121110) and *TGFβR2* (rs6550005) was significantly associated with the development of preeclampsia in American Caucasian women, while genetic variation in *TGFβ1* (rs4803455, rs4803457), *TGFβR1* (rs10739778), and *TGFβR2* (rs6550005, rs1346907, rd877572) was significantly associated with the development of preeclampsia in African American women [24]. For this original study we defined the preeclampsia phenotype based on the presence of hypertension, proteinuria, and hyperuricemia, and normotensive controls were 1:1 frequency matched to preeclampsia cases on ancestry, age, and parity.

The purpose of this current study was to [1] validate our genetic association findings from the American Caucasian cohort (PEPP cohort) in a well characterized Caucasian cohort from Norway and a well characterized White Hispanic cohort from Southern California and [2] explore the *ENG* pathway for new associations in these two cohorts.

## 2 Materials and methods

### 2.1 Study populations

De-identified clinical data and DNA samples were obtained from the Oslo Pregnancy Biobank and the “Pilot Study of Novel Candidate Genes for Preeclampsia” research study conducted at the University of Southern California, for Norwegian and Latina cohort participants, respectively.

Participants of the Oslo Pregnancy Biobank were women with singleton pregnancies who delivered via elective cesarean section between the years 2001 and 2008. Women with a history of chronic hypertension, renal disease, diabetes, or other chronic diseases were excluded. Preeclampsia was defined as new-onset hypertension and proteinuria after gestational week 20, in accordance with the American College of Obstetricians and Gynecologists 2002 criteria that were in place when the study was conducted [25]. New-onset hypertension was defined as systolic blood pressure (SBP) ≥140 mmHg or diastolic blood pressure (DBP) ≥90 mmHg after 20 weeks’ gestation, measured on ≥two occasions at least six hours apart in a previously normotensive woman. Proteinuria was defined as ≥0.3 g in a 24 h specimen or a urine dipstick ≥ 1+ on ≥ two midstream urine samples collected 6 h apart, in the absence of a urinary tract infection. Severe preeclampsia was defined as SBP ≥ 160 or DBP ≥ 110 on ≥ two occasions that were at least six hours apart (in accordance with the criteria outlined by the American College of Obstetricians and Gynecologists in 2002) [25]. HELLP syndrome was defined by the presence of hemolysis, elevated liver enzymes, and low platelets in the participants with preeclampsia. Partial HELLP was diagnosed when only two of the three signs were present. Normotensive (control) pregnancies were defined as those pregnancies in which a woman’s blood pressure did not reach 140/90 mmHg and she did not develop proteinuria. For this validation study, all control participants delivered appropriate for gestational age infants at term. Based on self-reported country of birth and names, all Norwegian participants included in this current study were categorized as White. The Oslo Pregnancy Biobank was approved by the Regional Committee of Medical Research Ethics in Eastern Norway, and use of DNA samples for the current study was approved by the University of Pittsburgh Institutional Review Board (IRB).

Participants of the “Pilot Study of Novel Candidate Genes for Preeclampsia” case-control research study (University of Southern California) were retrospectively recruited from delivery logs at the Los Angeles County and University of Southern California Women’s and Children’s Hospital or during their postpartum stay at the Women’s and Children’s Hospital between the years of 1999 and 2008. Women with lupus, chronic renal disease, multiple gestation, or sickle cell disease/trait were excluded from participation. Preeclampsia was also defined in alignment with the blood pressure and proteinuria criteria outlined by the American College of Obstetricians and Gynecologists in 2002 (please see criteria above) [25]. Severe preeclampsia was defined as SBP ≥ 160 mmHg or DBP ≥ 110 mmHg on ≥ two occasions at least 6 h apart AND proteinuria ≥ 500 mg/dL in a 24-h specimen or +3 on a urine dipstick. HELLP syndrome was defined by the presence of hemolysis (abnormal peripheral smear or LDH ≥ 600), elevated liver enzymes (ALT and/or AST ≥ 70), and low platelets (≤100,000). Partial HELLP was diagnosed when only two of the three signs were present. For this replication study, all participants with HELLP or partial HELLP also met the preeclampsia criteria. Uncomplicated pregnancies were defined as those pregnancies without significant hypertension. Based on self-report, all 175 Latina participants included in the current study were categorized as Hispanic Whites. The “Pilot Study of Novel Candidate Genes for Preeclampsia” research study was approved by the University of Southern California IRB, and use of the DNA samples in the current study was approved by the University of Pittsburgh IRB.

### 2.2 Genotyping methods

DNA aliquots obtained from the Norwegian cohort participants were extracted from EDTA peripheral blood samples using the MagNA Pure LC DNA Isolation Kit (Roche Diagnostics GmbH, Mannheim, Germany). DNA aliquots obtained from the Latina cohort participants were extracted from either peripheral blood samples or buccal swabs using the QIAamp DNA mini kit (Qiagen, Valencia, CA, USA), saliva samples using the Oragene kit (DNA Genotek, Ontario, Canada), or mouthwash samples using a phenyl-chloroform protocol previously described [26]. The genomic DNA aliquots provided by both the Norwegian and Latina cohorts were whole genome amplified using the GE™ Healthcare illustra™ GenomiPhi V2 DNA Amplification kit (GE Healthcare Life Sciences, Little Chalfont, United Kingdom). Methods for polymorphism selection, genotyping (iPLEX® Gold-SNP Genotyping assay (Sequenom® Inc, San Diego, CA; now Agena Bioscience, San Diego, CA), and data reliability checks are fully described by Bell et al. (2013) [24] (Table 1). The UCSC Genome Browser was used to identify tSNP positions within the genome (Human Assembly Dec. 2013 (GRCh38/hg38) [27]. We also used databases (UCSC Genome Browser, dbSNP) and conducted a literature search that combined gene name AND rs number as keywords (e.g., *TGFβR2* AND rs11129420) to learn about the potential function of the tSNPs that were significantly associated with pregnancy outcome in the multivariate models.

**Table 1:** Tagging and potentially functional single nucleotide polymorphisms assayed.

Candidate Gene	SNP rs numbers
Endoglin (*ENG*)	rs10987746, rs10819309, rs10760505, **rs11792480**, **rs10121110**
Transforming growth factor beta 1 (*TGFβ1*)	rs8179181, rs4803455, rs11466314, rs1800469, rs1800468, rs4803457
Transforming growth factor beta 1 receptor (*TGFβR1*)	rs6478974, rs10739778, rs420549
Activin receptor like kinase 1 (*ALK1*)	rs3759178, rs11169953, rs706819
Transforming growth factor beta receptor 2 (*TGFβR2*)	rs3087465, **rs6550005**, rs11129420, rs6802220, rs17025785, rs4522809, rs4955212, rs5020833, rs6809777, rs12487185, rs11924422, rs13083813, rs13075948, rs1155708, rs13086588, rs2082224, rs1078985, rs1036097, rs995435, rs6792117, rs749794, rs3773640, rs3773644, rs3773645, rs3773652, rs2043136, rs1346907, rs876688, rs877572, rs9843942, rs3773663, rs744751

Abbreviations: SNP, single nucleotide polymorphism. Notes: Bolded SNPs indicate the high priority SNPs analyzed in Tier 1 of this study. Underlined SNPs indicate the SNPs that were not successfully genotyped with iPLEX®.

### 2.3 Statistical analysis

Demographic and clinical characteristics were compared between cases and controls using parametric and non-parametric tests (e.g., Student’s *t*-test, Fisher’s exact test). For the main analyses, we utilized a tiered analytic approach. For the first analytic tier, we used Chi-square or Fisher’s exact tests (univariate analysis) and multivariate regression models to evaluate three tSNPs that were found to be significantly associated with pregnancy outcome in our previous study (rs11792480, rs10121110, rs6550005). In the second analytic tier, we evaluated the remaining tSNPs that had been genotyped. Univariate analysis with either Chi-square or Fisher’s exact tests was conducted to initially explore the relationship between pregnancy outcome and each tSNP. A p-value of p ≤ 0.15 was considered as potentially important. Multiple logistic regression models were then used to assess the relationship between potentially important tSNPs and pregnancy outcome while controlling for maternal age at delivery, infant sex, parity, self-reported smoking status during the index pregnancy, and pre-pregnancy body mass index (BMI). The adjusted odds ratio, 95% confidence interval, and the p-value of the tSNPs were reported. The overall goodness-of-fit of the logistic models were assessed by the Hosmer-Lemeshow test. We did not correct for multiple comparisons. Power analysis revealed that a sample size of 140 achieves 80% power to detect moderate effect size (W) of 0.2368 using a 1 degree of freedom Chi-square Test with a significance level (alpha) of 0.05.

In addition, we carried out a sub-group analysis to explore the association between tSNP genotype distributions and early vs. late-onset preeclampsia (e.g., preterm vs. term preeclampsia). We used gestational age at delivery as a surrogate marker for preeclampsia onset (early-onset preeclampsia: delivering at < 37.0 weeks’ gestation; late-onset preeclampsia: delivering at ≥37.0 weeks’ gestation) and compared. tSNP genotype distributions for each preeclampsia subgroup to the healthy control group using Fisher’s exact tests.

## 3 Results

### 3.1 Associations between *ENG* (rs11792480, rs10121110) and *TGFβR2* (rs6550005) tSNPs and preeclampsia not replicated in Norwegian and Latina cohorts

The demographic and clinical characteristics for the Norwegian and Latina cohorts are presented in Table 2, Table 3, respectively. In our previous genetic association study, we found that variation in two endoglin tSNPs (rs11792480, rs10121110) was associated with susceptibility to/protection from preeclampsia in an American Caucasian cohort, while variation in a *TGFβR2* tSNP (rs6550005) was associated with preeclampsia in both an American Caucasian and an African American cohort. We found additional tSNPs that were associated with preeclampsia in the African American cohort that were not found to be associated with preeclampsia in the American Caucasian cohort, but here we focused on tSNPs that were associated with preeclampsia in the American Caucasian cohort given that our replication cohorts were not of African ancestry.

**Table 2:** Demographic and clinical characteristics for Norwegian cohort.

Characteristic	Cases (n = 77)	Controls (n = 63)	p
Maternal age at delivery, years [mean ± SD]	31.8 ± 4.7	32.0 ± 4.6	.75
Nulliparous [n (%)]	48 (62.3%)	35 (55.6%)	.42
Gestational age at delivery (weeks) [median (min-max)]	33.9 (24.9–39.4)	38.7 (37.1–41.9)	<.001
Pre-pregnancy BMI (kg/m^2^) [median (min-max)]	23.7 (18.9–41.1)	22.3 (17.4–30.7)	.004
Smoked during pregnancy [n (%)]	8 (10.4%)	6 (9.5%)	.87

Abbreviations: BMI (kg/m^2^), body mass index (kilograms/meters squared). Notes: Pre-pregnancy BMI includes BMI pre-pregnancy or within the first trimester of pregnancy.

**Table 3:** Demographic and clinical characteristics for Latina cohort.

Characteristic	Cases (n = 69)	Controls (n = 106)	p-value
Maternal age at delivery, years [median (min–max)]	26.0 (17.0, 45.0)	25.0 (15.0, 42.0)	.3
Nulliparous (n, %)	31 (44.9%)	38 (35.8%)	.2
Gestational age at delivery (weeks) [median (min–max)]	37.0 (25.0, 41.0)	40.0 (37.0, 42.0)	<.001
Pre-pregnancy BMI (kg/m^2^)* [median (min–max)]	26.8 (19.6, 39.6)	24.2 (17.0, 41.8)	.02
Smoked during pregnancy** (n, %)	7 (10.9%)	4 (4.0%)	.08

Abbreviations: BMI (kg/m^2^), body mass index (kilograms/meters squared).

*Notes*:

* n = 11 controls and n = 2 cases were missing pre-pregnancy BMI values.

** n = 6 controls and n = 5 cases were missing values on smoking during pregnancy.

In both the Norwegian and Latina cohorts, associations between each of the three tSNPs of interest (*ENG*: rs11792480, rs10121110; *TGFβR2*: rs6550005) and preeclampsia status were not replicated in both the unadjusted and adjusted analyses (p’s > 0.05) (Refer to Supplemental Materials for tSNP/SNP genotype distributions). These associations were also non-significant in our exploratory subgroup analyses of early and late-onset preeclampsia.

### 3.2 Exploratory analysis provides additional support for the *ENG* pathway’s involvement in preeclampsia

Six intronic tSNPs in two *ENG* pathway candidate genes were found to be significantly associated with preeclampsia status in the Norwegian cohort, *TGFβR1*(rs6478974) and *TFFβR2*(rs11129420, rs6802220, rs1155708, rs3773640, rs3773663) (Table 4) (Refer to Supplemental Materials for tSNP/SNP genotype distributions).

**Table 4:** Multivariate logistic regression results in the Norwegian cohort: *ENG* Pathway Candidate Gene tSNPs Associated (p < .05) with Pregnancy Outcome.

Gene/tSNP	Genotype Distributions		HWE*	Genotype Comparisons	aOR [95% CI]**	p
	Cases: n (%)	Controls: n (%)				
TGFβR1(ALK5)/rs6478974 MAF: T = 0.46	AA: 16 (20.8)	AA: 23 (36.5)	NS	TA vs AA	2.38 [1.007–5.60]	0.05
	TT: 16 (20.8)	TT: 13 (20.6)				
	TA: 45 (58.4)	TA: 27 (42.9)				

*TGFβR2*/rs11129420 MAF: T = 0.48	AA: 12 (16.4)	AA: 18 (29.0)	0.02***	TT vs AA	5.01 [1.39–18.01]	0.01
	TT: 16 (21.9)	TT: 8 (12.9)		TA + TT vs AA	2.48 [1.001–6.17]	0.05
	TA: 45 (61.6)	TA: 36 (58.1)				

*TGFβR2*/rs6802220 MAF: A = 0.43	GG: 24 (32.9)	GG: 14 (22.6)	0.04***	AA vs. GG	0.17 [0.05–0.64]	0.009
	AA: 6 (8.2)	AA: 13 (21.0)		AA vs. AG + GG	0.27 [0.09–0.85]	0.02
	AG: 43 (58.9)	AG: 35 (56.5)				

*TGFβR2*/rs1155708 MAF: A = 0.40	GG: 32 (43.8)	GG: 20 (32.3)	NS	AA vs. GG	0.27 [0.07–0.99]	0.05
	AA: 5 (6.8)	AA: 11 (17.7)				
	GA: 36 (49.3)	GA: 31 (50.0)				

*TGFβR2*/rs3773640 MAF: T = 0.25	AA: 42 (57.5)	AA: 33 (54.1)	NS	AA vs. TT	9.86 [1.04–93.33]	0.05
	TT: 1 (1.4)	TT: 6 (9.8)		AT vs. TT	13.17 [1.31–132.21]	0.03
	AT: 30 (41.1)	AT: 22 (36.1)		AT + AA vs. TT	10.73 [1.15–100.12]	0.04

*TGFβR2*/rs3773663 MAF: G = 0.49	GG: 19 (26.0)	GG: 14 (22.6)	NS	AG vs AA	3.28 [1.28–8.36]	0.01
	AA: 14 (19.2)	AA: 22 (35.5)		AG + GG vs AA	3.08 [1.27–7.43]	0.01
	AG: 40 (54.8)	AG: 26 (41.9)				

Abbreviations: tSNP, tagging single nucleotide polymorphism; MAF, minor allele frequency; HWE, Hardy-Weinberg Equilibrium; aOR, adjusted odds ratio; CI, confidence interval; NS, non-significant (p > .05).

*Notes*:

* HWE assessed with Chi-square or Exact tests.

** The reference group for Pregnancy Outcome is the Case group. The multivariate logistic regression models were adjusted for maternal age, parity, pre-pregnancy BMI, smoking, and infant sex.

*** HWE was also assessed separately in cases and controls. The control group was in HWE while the case group was out of HWE.

Our exploratory subgroup findings suggest that certain tSNPs in two *ENG* pathway candidate genes were also found to be significantly associated with preeclampsia in the Latina cohort, *ALK1*(rs706819, 3′ UTR variant) and *TGFBR2*(rs984394, intronic) (Table 5). Because tSNP genotype distributions may differ by preeclampsia subgroup, we also compared preterm and term preeclampsia distributions to control distributions.

**Table 5:** Multivariate logistic regression results in the Latina cohort: *ENG* pathway Candidate gene tSNPs associated (p < .05) with pregnancy outcome.

Gene/tSNP	Genotype Distributions		HWE*	Genotype Group Comparisons	aOR [95% CI]**	p
	Cases: n(%)	Controls: n(%)				
*ALK1*/rs706819 MAF: G = 0.48	GG: 24 (35.3) AA: 21 (30.9) GA: 23 (33.8)	GG: 19 (18.1) AA: 33 (31.4) GA: 53 (50.5)	NS	GG vs GA GG vs AA + GA	3.59 [1.51–8.53] 2.62 [1.20–5.72]	0.004 0.02

*TGFβR2*/rs9843942 MAF: G = 0.44	AA: 29 (42.0) GG: 10 (14.5) GA: 30 (43.5)	AA: 29 (28.2) GG: 27 (26.2) GA: 47 (45.6)	NS	GG vs GA GG vs AA GG vs GA + GG	0.37 [0.14–1.0] 0.27 [0.096–0.76] 0.32 [0.13–0.83]	0.05 0.01 0.02

Abbreviations: tSNP, tagging single nucleotide polymorphism; MAF, minor allele frequency; HWE, Hardy-Weinberg Equilibrium; aOR, adjusted odds ratio; CI, confidence interval; NS, non-significant (p > .05).

*Notes*:

* HWE assessed with Chi-square or Exact tests.

** The reference group for Pregnancy Outcome is the Case group. The multivariate logistic regression models were adjusted for maternal age, parity, pre-pregnancy BMI, smoking, and infant sex.

## 4 Discussion

Preeclampsia is a heterogeneous disorder of pregnancy whose etiology may stem from a variety of underlying mechanisms. Women who develop early-onset and late-onset preeclampsia differ on average on several aspects, including their risk of future cardiovascular disease [28], but a dysfunctional placenta is seen as a major common pathophysiological pathway for triggering the maternal syndrome [29]. Some of the proposed mechanisms include defects in placental implantation, oxidative stress, a susceptible maternal constitution, and anti-angiogenic/angiogenic factor imbalance stemming from a dysfunctional placenta [30]. Endoglin and its soluble form, sENG, are angiogenesis-modulatory factors that are thought to have a role in the development of preeclampsia, and associations have been found at the gene, mRNA, and protein level. We have previously shown that genetic variants in the *ENG* pathway are associated with an increased susceptibility to preeclampsia in both American Caucasian and African American women [24]. Others have shown that mRNA expression of ENG is increased in the placenta, maternal blood (cellular and non-cellular components), and maternal microvascular endothelial cells [13], [14], [15], [16], [17], [18], [19], [20] while levels of sENG protein in the maternal circulation are elevated weeks to months before overt disease in women with preeclampsia [21], [22]. Although the evidence to support the alterations in *ENG* mRNA expression and circulating sENG protein levels is well documented across multiple studies, few studies have explored the role of *ENG* pathway genetic variation in preeclampsia development.

In this study we investigated the association between maternal genetic variation in the *ENG* pathway and susceptibility to/protection from preeclampsia in independent, well-characterized preeclampsia cohorts. We first sought to validate our genetic association findings from an American Caucasian preeclampsia cohort (*ENG* tSNPs rs11792480, rs10121110; *TGFβR2* tSNP rs6550005) in Norwegian and Latina cohorts. We further explored the other gene candidates from the *ENG* pathway for new genetic associations with preeclampsia in these cohorts. We were unable to validate/replicate the SNP-specific previous findings in these independent cohorts. However, we identified other associations between *ENG* pathway candidate genes and preeclampsia in the Norwegian (*TGFβR1[ALK5]*, *TGFβR2*) and Latina (*ALK1*, *TGFβR2*) cohorts in our adjusted models. We have previously demonstrated that variation in *TGFβR1[ALK5]* was associated with preeclampsia in an African American cohort [24]. Moreover, we have demonstrated that genetic variation in *TGFβR2* is associated with susceptibility to/protection from preeclampsia in all of the populations we have studied. Although the *TGFβR2* tSNPs that were associated with a particular cohort may differ, these findings, along with the other associations in the Norwegian and Latina cohorts, provide additional evidence to support the ENG pathway’s role/involvement in the development of preeclampsia.

### 4.1 Failure to validate/replicate associations between genetic variation in *ENG* and *TGFβR2*

Several potential explanations could account for our inability to validate our previous results in independent cohorts. First, these cohorts may not have been sufficiently powered to detect statistically significant associations when they truly existed. Second, tSNPs that were selected based on Caucasian ancestry (CEU population-European descendants living in Utah) may not be as informative in the Norwegian and Latina cohorts if the haploblocks tagged by the tSNPs differ by ancestry. Finally, differences in the definition of preeclampsia phenotype may underlie our failure to validate our previous results. In the original cohort, a diagnosis of preeclampsia was based on hypertension, proteinuria, and hyperuricemia. Hyperuricemia was included in the original cohort’s research definition as a way to identify a more homogeneous and potentially more severe form of preeclampsia. In both the Norwegian and Latina cohorts, hyperuricemia was not included as a criterion for the preeclampsia phenotype. Given the heterogeneous nature of preeclampsia, these cohorts may represent different preeclampsia subtypes with differing etiologies.

### 4.2 Additional evidence to support the ENG pathway’s involvement in preeclampsia

Genetic variation in *TGFβR1[ALK5]* and *TGFβR2* was associated with susceptibility to/protection from preeclampsia in the Norwegian cohort, while genetic variation in *ALK1* and *TGFβR2* was associated with susceptibility to/protection from preeclampsia in the Latina cohort. Seven of the eight significantly associated tSNPs (Table 5; rs6478974, rs11129420, rs680220, rs1155708, rs3773640, rs3773663, rs9843942) are intronic, while the other SNP was located in the 3′ UTR of the gene (rs706819). Of the eight tSNPs, we were only able to locate a small amount of literature on *TGFβR1* tSNP rs6478974, which has been associated with endometrial and gastric cancer in other populations [31], [32].

TGFβR1[ALK5] and ALK1 are type 1 receptors of the TGFβ signaling cascade, which form a heteromeric complex with TGFβR2, the type 2 receptor of the TGFβ signaling cascade. In most cells, TGFβ signals via ALK5, but TGFβ can also signal via ALK1 in endothelial cells. The *TGFβ1* ligand binds to these complexes, and propagation of the cell signal is involved in the control of a variety of processes including cellular proliferation, migration, and differentiation [10].

In vitro research has demonstrated that *TGFβ1* is a negative regulator of trophoblast invasion toward the invasive phenotype [33], [34], [35], [36], and that endoglin is needed to facilitate this inhibitory effect [11], [12]. Shallow trophoblast invasion and failed remodeling of the spiral arteries, affects mostly the early-onset subtype of preeclampsia [37], [38], [39], [40]. Furthermore, implantation and placental development is dependent on the successful interaction between fetal/placental and maternal/decidual factors [34]. As such, it is plausible that maternal or fetal genetic variation in any of the members of the ENG/TGFβ pathway could impact TGFβ signaling and the regulation of trophoblast invasion. Moreover, it is possible that maternal genetic variation in this pathway could also impact systemic endothelial function in the mother, as this pathway is involved in the maintenance of vascular homeostasis, including vasomotor tone [10], [41], [42].

### 4.3 Study limitations

In addition to small sample size and differences in ancestry that were previously discussed, there were several other limitations associated with this study. First, the cohorts’ sample sizes were further reduced when we carried out our exploratory preeclampsia subgroup analyses, which limited our ability to adequately evaluate preeclampsia subgroups. Second, we did not adjust for multiple comparisons and therefore we cannot rule out the potential for type 1 error. As such, follow-up studies with larger sample sizes, including larger preeclampsia subgroups, will be needed to validate these results. Third, we were unable to successfully genotype three SNPs (*TGFβ1* rs1800468; *TGFβR2* rs1078985 and rs995435).

## 5 Summary

Overall, our results provide further support for the involvement and investigation of the endoglin pathway in preeclampsia, yet additional research with larger sample sizes, including larger samples of different preeclampsia subgroups, is needed. Due to the relatively low frequency of preeclampsia, there is a need for investigators across the globe to collaborate and share data/biological samples with each other so that we can continue to put preeclampsia’s puzzle pieces together.
