# Supplementary material for: Endoglin pathway genetic variation in preeclampsia: A validation study in Norwegian and Latina cohorts
Source: Pregnancy Hypertens. 2018 Apr;12:144–9. doi: 10.1016/j.preghy.2017.10.005 (PMC5995147; doi:10.1016/j.preghy.2017.10.005)
Supplement: Supplementary data 1 [file mmc1.docx]

**Endoglin Pathway Genetic Variation in Preeclampsia: A Validation Study in Norwegian and Latina Cohorts**

**SUPPLEMENTARY MATERIAL**

Mandy J. Schmella, PhD, RN^a^; James M. Roberts, MD^b-e;m^; Yvette P. Conley, PhD^a,f^; Dianxu Ren, MD, PhD^g^; Gro L. Storvold, PhD^h-i^; Sue A. Ingles, DPH^j-k^; Melissa L. Wilson, MPH, PhD^k;m^; Anne Catherine Staff, MD, PhD^h-i;m^; Carl A. Hubel, PhD^b-c;l^

^a^Department of Health Promotion and Development (School of Nursing); University of Pittsburgh; Pittsburgh, Pennsylvania, USA

^b^Department of Obstetrics, Gynecology, & Reproductive Sciences (School of Medicine); University of Pittsburgh, Pennsylvania, USA

^c^Magee-Womens Research Institute; Pittsburgh, Pennsylvania, USA

^d^Department of Epidemiology (Graduate School of Public Health); University of Pittsburgh; Pittsburgh, Pennsylvania, USA

^e^Clinical and Translational Science Institute; University of Pittsburgh; Pittsburgh, Pennsylvania, USA

^f^Department of Human Genetics (Graduate School of Public Health); University of Pittsburgh; Pittsburgh, Pennsylvania, USA

^g^Department of Health and Community Systems (School of Nursing); University of Pittsburgh; Pittsburgh, Pennsylvania, USA

^h^Faculty of Medicine, University of Oslo; Oslo, Norway

^i^Division of Obstetrics and Gynaecology; Oslo University Hospital; Oslo, Norway

^j^Department of Obstetrics and Gynecology (Keck School of Medicine); University of Southern California; Los Angeles, California, USA

^k^Department of Preventive Medicine (Keck School of Medicine); University of Southern California; Los Angeles, California, USA

^l^Department of Environmental and Occupational Health (Graduate School of Public Health); University of Pittsburgh; Pittsburgh, Pennsylvania, USA

^m^Global Pregnancy Collaboration

**Polymorphism Selection, Genotyping Methods, and Genotype Data Reliability Checks**

As previously described,^1^ a tagging single nucleotide polymorphism (tSNP) approach was used to assess genetic variability in the five endoglin pathway genes (*ENG*, *TGFβ1*, *TGFβR1*, *ALK1*, and *TGFβR2*). The same iPLEX® Gold-SNP Genotyping assay (Sequenom® Inc, San Diego, CA; now Agena Bioscience, San Diego, CA) designed to assess the 49 SNPs across the five candidate genes in the original study^1^ was also used in this study (Table 1).

Global call rates for each of the 49 SNPs were assessed using the raw genotype data. Three SNPs with global call rates of < 90% were omitted from the analysis (*TGFβR2* tSNPs rs995435 and rs1078985, *TGFβ1* potentially functional SNP rs1800468). Genotype reliability was assessed via Hardy-Weinberg Equilibrium (HWE) checks (R statistics package used). Tagging SNPs found to be out of HWE (p>0.05) in either the Norwegian or Latina cohorts, were further assessed for HWE in cases and controls separately. We concluded that the violation of HWE in the entire cohort, either Norwegian or Latina, was either due to enrichment for preeclampsia in the case groups or non-preeclampsia in the control groups, and not due to genotyping error.

Reference:

^1^Bell MJ, Roberts JM, Founds SA, Jeyabalan A, Terhorst L, Conley YP. Variation in endoglin pathway genes is associated with preeclampsia: a case-control candidate gene association study. BMC pregnancy and childbirth. 2013;13:82.

**Table S1. Norwegian Cohort tSNP/SNP Genotype Distributions (N=77 cases and N=63 controls)**

| Gene  SNP | Genotype Counts (%) Cases | Genotype Counts  (%) Controls | HWE | Bivariate Associations  (p value) |
| --- | --- | --- | --- | --- |
| *ALK5 (TGFBR1)* |  |  |  |  |
| rs6478974 | AA: 16 (20.8)  TT: 16 (20.8)  TA: 45 (58.4) | AA: 23 (36.5)  TT: 13 (20.6)  TA: 27 (42.9) | NS | AA vs TT vs TA: 0.09  AA vs TA + TT: 0.04  TA + AA vs TT: 0.98 |
| rs10739778 | AA: 33 (42.9)  CC: 10 (13.0)  CA: 34 (44.2) | AA: 28 (44.4)  CC: 5 (7.9)  CA: 30 (47.6) | NS | AA vs CC vs CA: 0.63  AA vs CA + CC: 0.85  CA + AA vs CC: 0.34 |
| rs420549 | GG: 56 (72.7%)  CC: 0 (0.0%)  GC: 21 (27.3%) | GG: 44 (71.0%)  CC: 2 (3.2%)  GC: 16 (25.8%) | NS | GG vs CC vs GC: 0.36  CC vs GC + GG: 0.20  GC + CC vs GG: 0.82 |
| *ALK1* |  |  |  |  |
| rs3759178 | TT: 29 (37.7)  GG: 8 (10.4)  GT: 40 (51.9) | TT: 24 (38.1)  GG: 3 (4.8)  GT: 36 (57.1) | 0.02 | TT vs GG vs GT: 0.46  GG vs GT + TT: 0.34  GT + GG vs TT: 0.96 |
| rs11169953 | CC: 35 (46.1)  TT: 8 (10.5)  CT: 33 (43.4) | CC: 25 (39.7)  TT: 6 (9.5)  CT: 32 (50.8) | NS | CC vs TT vs CT: 0.68  CC vs CT + TT: 0.45  CT + CC vs TT: 0.85 |
| rs706819 | GG: 43 (55.8)  AA: 6 (7.8)  GA: 28 (36.4) | GG: 29 (46.0)  AA: 2 (3.2)  GA: 32 (50.8) | NS | GG vs AA vs GA: 0.19  AA vs GA + GG: 0.30  GA + AA vs GG: 0.25 |
| *TGFB1* |  |  |  |  |
| rs8179181 | CC: 50 (72.5)  TT: 2 (2.9)  CT: 17 (24.6) | CC: 37 (62.7)  TT: 6 (10.2)  CT: 16 (27.1) | NS | CC vs TT vs CT: 0.22  CC vs CT + TT: 0.24  CT + CC vs TT: 0.14 |
| rs4803455 | CC: 21 (27.3)  AA: 18 (23.4)  CA: 38 (49.4) | CC: 11 (17.5)  AA: 11 (17.5)  CA: 41 (65.1) | NS | CC vs AA vs CA: 0.17  AA vs CA + CC: 0.39  CA + AA vs CC: 0.17 |
| rs11466314 | GG: 77 (100) | GG: 63 (100) | NS | ---------------------------- |
| rs1800469 | CC: 39 (50.6)  TT: 10 (13.0)  CT: 28 (36.4) | CC: 34 (54.0)  TT: 4 (6.3)  CT: 25 (39.7) | NS | CC vs TT vs CT: 0.43  CC vs CT + TT: 0.70  CT + CC vs TT: 0.19 |
| rs4803457 | CC: 31 (40.8)  TT: 14 (18.4)  CT: 31 (40.8) | CC: 26 (41.3)  TT: 6 (9.5)  CT: 31 (49.2) | NS | CC vs TT vs CT: 0.30  CC vs CT + TT: 0.95  CT + CC vs TT: 0.14 |
| *ENG* |  |  |  |  |
| rs10987746 | TT: 20 (26.0)  CC: 14 (18.2)  TC: 43 (55.8) | TT: 19 (30.6)  CC: 10 (16.1)  TC: 33 (53.2) | NS | TT vs CC vs TC: 0.82  CC vs TC + TT: 0.75  TC + CC vs TT: 0.54 |
| rs10819309 | GG: 27 (35.1)  AA: 11 (14.3)  GA: 39 (50.6) | GG: 20 (31.7)  AA: 13 (20.6)  GA: 30 (47.6) | NS | GG vs AA vs GA: 0.61  AA vs GA + GG: 0.32  GA + AA vs GG: 0.68 |
| rs10760505 | CC: 30 (39.0)  TT: 10 (13.0)  CT: 37 (48.1) | CC: 25 (39.7)  TT: 9 (14.3)  CT: 29 (46.0) | NS | CC vs TT vs CT: 0.96  CC vs CT + TT: 0.93  CT + CC vs TT: 0.82 |
| rs11792480 | GG: 36 (46.8)  AA: 5 (6.5)  AG: 36 (46.8) | GG: 32 (50.8)  AA: 7 (11.1)  AG: 24 (38.1) | NS | GG vs AA vs AG: 0.45  AA vs AG + GG: 0.33  AG + AA vs GG: 0.63 |
| rs10121110 | AA: 39 (50.6)  GG: 10 (13.0)  AG: 28 (36.4) | AA: 28 (45.9)  GG: 10 (16.4)  AG: 23 (37.7) | NS | AA vs GG vs AG: 0.80  AA vs AG + GG: 0.58  AG + AA vs GG: 0.57 |
| *TGFBR2* |  |  |  |  |
| rs3087465 | GG: 42 (54.5)  AA: 4 (5.2)  AG: 31 (40.3) | GG: 31 (49.2)  AA: 7 (11.1)  AG: 25 (39.7) | NS | GG vs AA vs AG: 0.42  AA vs AG + GG: 0.22  AG + AA vs GG: 0.53 |
| rs6550005 | GG: 41 (57.7)  AA: 2 (2.8)  GA: 28 (39.4) | GG: 35 (56.5)  AA: 1 (1.6)  GA: 26 (41.9) | NS | GG vs AA vs GA: 0.94  AA vs GA +GG: > 0.99  GA +AA vs GG: 0.88 |
| rs11129420 | AA: 12 (16.4)  TT: 16 (21.9)  TA: 45 (61.6) | AA: 18 (29.0)  TT: 8 (12.9)  TA: 36 (58.1) | 0.02 | AA vs TT vs TA: 0.14  AA vs TA + TT: 0.08  TA + AA vs TT: 0.17 |
| rs6802220 | GG: 24 (32.9)  AA: 6 (8.2)  AG: 43 (58.9) | GG: 14 (22.6)  AA: 13 (21.0)  AG: 35 (56.5) | 0.04 | GG vs AA vs AG: 0.08  AA vs AG + GG: 0.03  AG + AA vs GG: 0.19 |
| rs17025785 | TT: 26 (35.6)  CC: 5 (6.8)  TC: 42 (57.5) | TT: 21 (33.9)  CC: 6 (9.7)  TC: 35 (56.5) | 0.008 | TT vs CC vs TC: 0.83  CC vs TC + TT: 0.55  TC + CC vs TT: 0.83 |
| rs4522809 | TT: 18 (24.7)  CC: 18 (24.7)  CT: 37 (50.7) | TT: 14 (22.6)  CC: 9 (14.5)  CT: 39 (62.9) | NS | TT vs CC vs CT: 0.26  CC vs CT + TT: 0.14  CT + CC vs TT: 0.78 |
| rs4955212 | CC: 37 (51.4)  TT: 3 (4.2)  CT: 32 (44.4) | CC: 41 (66.1)  TT: 2 (3.2)  CT: 19 (30.6) | NS | CC vs TT vs CT: 0.21  CC vs CT + TT: 0.08  CT +CC vs TT: >0.99 |
| rs5020833 | CC: 37 (50.7)  GG: 3 (4.1)  CG: 33 (45.2) | CC: 25 (40.3)  GG: 4 (6.5)  CG: 33 (53.2) | NS | CC vs GG vs CG: 0.46  CC vs CG + GG: 0.23  CG + CC vs GG: 0.70 |
| rs6809777 | CC: 41 (56.2)  TT: 9 (12.3)  CT: 23 (31.5) | CC: 29 (49.2)  TT: 6 (10.2)  CT: 24 (40.7) | NS | CC vs TT vs CT: 0.55  CC vs CT + TT: 0.42  CT + CC vs TT: 0.70 |
| rs12487185 | AA: 36 (49.3)  GG: 4 (5.5)  GA: 33 (45.2) | AA: 27 (43.5)  GG: 8 (12.9)  GA: 27 (43.5) | NS | AA vs GG vs GA: 0.31  AA vs GA + GG: 0.50  GA + AA vs GG: 0.13 |
| rs11924422 | AA: 18 (24.7)  CC: 9 (12.3)  CA: 46 (63.0) | AA: 23 (37.1)  CC: 8 (12.9)  CA: 31 (50.0) | 0.04 | AA vs CC vs CA: 0.26  AA vs CA + CC: 0.12  CA + AA vs CC: 0.92 |
| rs13083813 | TT: 25 (34.2)  AA: 8 (11.0)  AT: 40 (54.8) | TT: 31 (50.0)  AA: 4 (6.5)  AT: 27 (43.5) | NS | TT vs AA vs AT: 0.16  AA vs AT + TT: 0.36  AT + AA vs TT: 0.06 |
| rs13075948 | CC: 35 (47.9)  TT: 7 (9.6)  CT: 31 (42.5) | CC: 35 (56.5)  TT: 9 (14.5)  CT: 18 (29.0) | NS | CC vs TT vs CT: 0.24  CC vs CT + TT: 0.32  CT + CC vs TT: 0.38 |
| rs1155708 | GG: 32 (43.8)  AA: 5 (6.8)  GA: 36 (49.3) | GG: 20 (32.3)  AA: 11 (17.7)  GA: 31 (50.0) | NS | GG vs AA vs GA: 0.10  AA vs GA + GG: 0.05  GA + AA vs GG: 0.17 |
| rs13086588 | TT: 32 (43.8)  GG: 6 (8.2)  GT: 35 (47.9) | TT: 22 (35.5)  GG: 7 (11.3)  GT: 33 (53.2) | NS | TT vs GG vs GT: 0.58  GG vs GT + TT: 0.55  GT + GG vs TT: 0.32 |
| rs2082224 | GG: 45 (61.6)  AA: 3 (4.1)  GA: 25 (34.2) | GG: 32 (51.6)  AA: 4 (6.5)  GA: 26 (41.9) | NS | GG vs AA vs GA: 0.50  AA vs GA + GG: 0.70  GA + AA vs GG: 0.24 |
| rs1036097 | GG: 19 (26.0)  AA: 16 (21.9)  GA: 38 (52.1) | GG: 20 (32.3)  AA: 8 (12.9)  GA: 34 (54.8) | NS | GG vs AA vs GA: 0.36  AA vs GA + GG: 0.17  GA + AA vs GG: 0.43 |
| rs6792117 | GG: 21 (28.8)  AA: 11 (15.1)  GA: 41 (56.2) | GG: 16 (25.8)  AA: 14 (22.6)  GA: 32 (51.6) | NS | GG vs AA vs GA: 0.53  AA vs GA + GG: 0.26  GA + AA vs GG: 0.70 |
| rs749794 | TT: 35 (45.5)  CC: 4 (5.2)  TC: 38 (49.4) | TT: 25 (39.7)  CC: 7 (11.1)  TC: 31 (49.2) | NS | TT vs CC vs TC: 0.40  CC vs TC + TT: 0.22  TC + CC vs TT: 0.49 |
| rs3773640 | AA: 42 (57.5)  TT: 1 (1.4)  AT: 30 (41.1) | AA: 33 (54.1)  TT: 6 (9.8)  AT: 22 (36.1) | NS | AA vs TT vs AT: 0.09  AA vs AT + TT: 0.69  AT + AA vs TT: 0.05 |
| rs3773644 | CC: 23 (31.5)  TT: 11 (15.1)  CT: 39 (53.4) | CC: 15 (24.2)  TT: 7 (11.3)  CT: 40 (64.5) | 0.02 | CC vs TT vs CT: 0.43  CC vs CT + TT: 0.35  CT + CC vs TT: 0.52 |
| rs3773645 | CC: 31 (42.5)  GG: 7 (9.6)  CG: 35 (47.9) | CC: 29 (47.5)  GG: 5 (8.2)  CG: 27 (44.3) | NS | CC vs GG vs CG: 0.84  CC vs CG + GG: 0.56  CG + CC vs GG: 0.78 |
| rs3773652 | AA: 27 (37.0)  GG: 10 (13.7)  AG: 36 (49.3) | AA: 18 (29.0)  GG: 9 (14.5)  AG: 35 (56.5) | NS | AA vs GG vs AG: 0.62  AA vs AG + GG: 0.33  AG + AA vs GG: 0.89 |
| rs2043136 | TT: 47 (64.4)  CC: 1 (1.4)  TC: 25 (34.2) | TT: 31 (50.0)  CC: 6 (9.7)  TC: 25 (40.3) | NS | TT vs CC vs TC: 0.05  CC vs TC + TT: 0.05  TC + CC vs TT: 0.09 |
| rs1346907 | CC: 21 (28.8)  TT: 14 (19.2)  CT: 38 (52.1) | CC: 24 (38.7)  TT: 12 (19.4)  CT: 26 (41.9) | NS | CC vs TT vs CT: 0.42  CC vs CT + TT: 0.22  CT + CC vs TT: 0.98 |
| rs876688 | GG: 27 (37.0)  AA: 13 (17.8)  GA: 33 (45.2) | GG: 27 (43.5)  AA: 8 (12.9)  GA: 27 (43.5) | NS | GG vs AA vs GA: 0.64  AA vs GA + GG: 0.43  GA + AA vs GG: 0.44 |
| rs877572 | GG: 21 (28.8)  CC: 14 (19.2)  CG: 38 (52.1) | GG: 24 (38.7)  CC: 10 (16.1)  CG: 28 (45.2) | NS | GG vs CC vs CG: 0.47  CC vs CG + GG: 0.64  CG + CC vs GG: 0.22 |
| rs9843942 | GG: 21 (28.8)  AA: 13 (17.8)  GA: 39 (53.4) | GG: 25 (40.3)  AA: 12 (19.4)  GA: 25 (40.3) | NS | GG vs AA vs GA: 0.28  AA vs GA + GG: 0.82  GA + AA vs GG: 0.16 |
| rs3773663 | GG: 19 (26.0)  AA: 14 (19.2)  AG: 40 (54.8) | GG: 14 (22.6)  AA: 22 (35.5)  AG: 26 (41.9) | NS | GG vs AA vs AG: 0.10  AA vs AG + GG: 0.03  AG + AA vs GG: 0.64 |
| rs744751 | CC: 29 (39.7)  TT: 8 (11.0)  TC: 36 (49.3) | CC: 28 (45.2)  TT: 9 (14.5)  TC: 25 (40.3) | NS | CC vs TT vs TC: 0.56  CC vs TC + TT: 0.52  TC + CC vs TT: 0.53 |

**Table S2. Latina Cohort tSNP/SNP Genotype Distributions (N=69 cases and N=106 controls)**

| Gene  SNP | Genotype Counts (%) Cases | Genotype Counts (%) Controls | HWE | Bivariate Associations (p value) |
| --- | --- | --- | --- | --- |
| *ALK5 (TGFBR1)* |  |  |  |  |
| rs6478974 | TT: 26 (38.2)  AA: 11 (16.2)  TA: 31 (45.6) | TT: 46 (43.8)  AA: 12 (11.4)  TA: 47 (44.8) | NS | TT vs AA vs TA: 0.60  AA vs TA + TT: 0.37  TA + AA vs TT: 0.47 |
| rs10739778 | AA: 31 (45.6)  CC: 9 (13.2)  CA: 28 (41.2) | AA: 53 (50.5)  CC: 9 (8.6)  CA: 43 (41.0) | NS | AA vs CC vs CA: 0.59  AA vs CA + CC: 0.53  CA + AA vs CC: 0.33 |
| rs420549 | GG: 56 (82.4)  CC: ---  GC: 12 (17.6) | GG: 90 (85.7)  CC: ---  GC: 15 (14.3) | NS | GG vs GC: 0.55 |
| *ALK1* |  |  |  |  |
| rs3759178 | TT: 24 (35.3)  GG: 10 (14.7)  GT: 34 (50.0) | TT: 38 (36.5)  GG: 14 (13.5)  GT: 52 (50.0) | NS | TT vs GG vs GT: 0.97  GG vs GT + TT: 0.82  GT + GG vs TT: 0.87 |
| rs11169953 | CC: 31 (46.3)  TT: 6 (9.0)  CT: 30 (44.8) | CC: 51 (49.5)  TT: 4 (3.9)  CT: 48 (46.6) | NS | CC vs TT vs CT: 0.39  CC vs CT + TT: 0.68  CT + CC vs TT: 0.19 |
| rs706819 | GG: 24 (35.3)  AA: 21 (30.9)  GA: 23 (33.8) | GG: 19 (18.1)  AA: 33 (31.4)  GA: 53 (50.5) | NS | GG vs AA vs GA: 0.02  AA vs GA + GG: 0.94  GA + AA vs GG: 0.01 |
| *TGFB1* |  |  |  |  |
| rs8179181 | CC: 56 (84.8)  TT: 1 (1.5)  CT: 9 (13.6) | CC: 84 (84.8)  TT: ---  CT: 15 (15.2) | NS | CC vs TT vs CT: 0.62  CC vs CT + TT: >0.99  CT + CC vs TT: 0.40 |
| rs4803455 | CC: 34 (50.0)  AA: 7 (10.3)  CA: 27 (39.7) | CC: 52 (50.0)  AA: 7 (6.7)  CA: 45 (43.3) | NS | CC vs AA vs CA: 0.68  AA vs CA + CC: 0.40  CA + AA vs CC: >0.99 |
| rs11466314 | GG: 68 (100) | GG: 105 (100) | NS | ----------------------------- |
| rs1800469 | CC: 15 (22.7)  TT: 17 (25.8)  CT: 34 (51.5) | CC: 25 (24.0)  TT: 23 (22.1)  CT: 56 (53.8) | NS | CC vs TT vs CT: 0.86  CC vs CT + TT: 0.84  CT + CC vs TT: 0.59 |
| rs4803457 | TT: 20 (30.3)  CC: 15 (22.7)  CT: 31 (47.0) | TT: 29 (28.2)  CC: 20 (19.4)  CT: 54 (52.4) | NS | TT vs CC vs CT: 0.77  CC vs CT + TT: 0.60  CT + CC vs TT: 0.76 |
| *ENG* |  |  |  |  |
| rs10987746 | TT: 25 (36.8)  CC: 15 (22.1)  TC: 28 (41.2) | TT: 29 (27.9)  CC: 24 (23.1)  TC: 51 (49.0) | NS | TT vs CC vs TC: 0.45  CC vs TC + TT: 0.88  TC + CC vs TT: 0.22 |
| rs10819309 | GG: 20 (29.9)  AA: 13 (19.4)  GA: 34 (50.7) | GG: 44 (42.3)  AA: 13 (12.5)  GA: 47 (45.2) | NS | GG vs AA vs GA: 0.20  AA vs GA + GG: 0.22  GA + AA vs GG: 0.10 |
| rs10760505 | CC: 26 (38.2)  TT: 12 (17.6)  CT: 30 (44.1) | CC: 37 (35.6)  TT: 19 (18.3)  CT: 48 (46.2) | NS | CC vs TT vs CT: 0.94  CC vs CT + TT: 0.72  CT + CC vs TT: 0.92 |
| rs11792480 | GG: 49 (72.1)  AA: 2 (2.9)  AG: 17 (25.0) | GG: 72 (69.2)  AA: 7 (6.7)  AG: 25 (24.0) | NS | GG vs AA vs AG: 0.65  AA vs AG + GG: 0.49  AG + AA vs GG: 0.69 |
| rs10121110 | AA: 38 (57.6)  GG: 5 (7.6)  AG: 23 (34.8) | AA: 58 (56.3)  GG: 11 (10.7)  AG: 34 (33.0) | NS | AA vs GG vs AG: 0.79  AA vs AG + GG: 0.87  AG + AA vs GG: 0.50 |
| *TGFBR2* |  |  |  |  |
| rs3087465 | GG: 37 (54.4)  AA: 5 (7.4)  AG: 26 (38.2) | GG: 57 (55.3)  AA: 6 (5.8)  AG: 40 (38.8) | NS | GG vs AA vs AG: 0.92  AA vs AG + GG: 0.76  AG + AA vs GG: 0.91 |
| rs6550005 | GG: 52 (75.4)  AA: 1 (1.4)  GA: 16 (23.2) | GG: 85 (82.5)  AA: 1 (1.0)  GA: 17 (16.5) | NS | GG vs AA vs GA: 0.47  AA vs GA + GG: >0.99  AA + GA vs GG: 0.25 |
| rs11129420 | AA: 23 (33.3)  TT: 14 (20.3)  TA: 32 (46.4) | AA: 38 (36.5)  TT: 13 (12.5)  TA: 53 (51.0) | NS | AA vs TT vs TA: 0.38  AA vs TA + TT: 0.67  TA + AA vs TT: 0.17 |
| rs6802220 | GG: 26 (37.7)  AA: 14 (20.3)  AG: 29 (42.0) | GG: 31 (29.8)  AA: 23 (22.1)  AG: 50 (48.1) | NS | GG vs AA vs AG: 0.56  AA vs AG + GG: 0.77  AG + AA vs GG: 0.28 |
| rs17025785 | TT: 26 (37.7)  CC: 12 (17.4)  TC: 31 (44.9) | TT: 29 (27.9)  CC: 28 (26.9)  TC: 47 (45.2) | NS | TC vs CC vs TC: 0.24  CC vs TC + TT: 0.15  TC + CC vs TT: 0.18 |
| rs4522809 | CC: 20 (29.0)  TT: 15 (21.7)  CT: 34 (49.3) | CC: 19 (18.3)  TT: 33 (31.7)  CT: 52 (50.0) | NS | CC vs TT vs CT: 0.16  CC vs CT + TT: 0.01  CT + CC vs TT: 0.15 |
| rs4955212 | CC: 25 (36.8)  TT: 10 (14.7)  CT: 33 (48.5) | CC: 45 (44.6)  TT: 11 (10.9)  CT: 45 (44.6) | NS | CC vs TT vs CT: 0.55  CC vs CT + TT: 0.31  CT + CC vs TT: 0.46 |
| rs5020833 | CC: 32 (46.4)  GG: 12 (17.4)  CG: 25 (36.2) | CC: 41 (39.4)  GG: 21 (20.2)  CG: 42 (40.4) | 0.02 | CC vs GG vs CG: 0.66  CC vs CG + GG: 0.36  CG + CC vs GG: 0.65 |
| rs6809777 | CC: 47 (71.2)  TT: 1 (1.5)  CT: 18 (27.3) | CC: 68 (65.4)  TT: ---  CT: 36 (34.6) | NS | CC vs TT vs CT: 0.27  CC vs CT + TT: 0.43  CT + CC vs TT: 0.39 |
| rs12487185 | AA: 35 (50.7)  GG: 9 (13.0)  GA: 25 (36.2) | AA: 41 (39.8)  GG: 19 (18.4)  GA: 43 (41.7) | NS | AA vs GG vs GA: 0.34  AA vs GA + GG: 0.16  GA + AA vs GG: 0.35 |
| rs11924422 | AA: 22 (31.9)  CC: 16 (23.2)  CA: 31 (44.9) | AA: 29 (27.9)  CC: 16 (15.4)  CA: 59 (56.7) | NS | AA vs CC vs CA: 0.26  AA vs CA + CC: 0.57  CA + AA vs CC: 0.20 |
| rs13083813 | TT: 23 (33.3)  AA: 16 (23.2)  AT: 30 (43.5) | TT: 34 (32.7)  AA: 13 (12.5)  AT: 57 (54.8) | NS | TT vs AA vs AT: 0.14  AA vs AT + TT: 0.07  AT + AA vs TT: 0.93 |
| rs13075948 | CC: 46 (66.7)  TT: 2 (2.9)  CT: 21 (30.4) | CC: 61 (58.7)  TT: 2 (1.9)  CT: 41 (39.4) | NS | CC vs TT vs CT: 0.48  CC vs CT + TT: 0.29  CT + CC vs TT: >0.99 |
| rs1155708 | GG: 29 (42.0)  AA: 11 (15.9)  GA: 29 (42.0) | GG: 45 (43.7)  AA: 16 (15.5)  GA: 42 (40.8) | NS | GG vs AA vs GA: 0.98  AA vs GA + GG: 0.94  GA + AA vs GG: 0.83 |
| rs13086588 | TT: 32 (47.1)  GG: 11 (16.2)  GT: 25 (36.8) | TT: 44 (42.3)  GG: 12 (11.5)  GT: 48 (46.2) | NS | TT vs GG vs GT: 0.42  GG vs GT + TT: 0.38  GT + GG vs TT: 0.54 |
| rs2082224 | GG: 35 (50.7)  AA: 7 (10.1)  GA: 27 (39.1) | GG: 49 (47.6)  AA: 9 (8.7)  GA: 45 (43.7) | NS | GG vs AA vs GA: 0.83  AA vs GA + GG: 0.76  GA + AA vs GG: 0.69 |
| rs1036097 | GG: 18 (26.1)  AA: 20 (29.0)  GA: 31 (44.9) | GG: 27 (26.0)  AA: 23 (22.1)  GA: 54 (51.9) | NS | GG vs AA vs GA: 0.55  AA vs GA + GG: 0.31  GA + AA vs GG: 0.99 |
| rs6792117 | AA: 23 (33.3)  GG: 13 (18.8)  GA: 33 (47.8) | AA: 39 (37.5)  GG: 18 (17.3)  GA: 47 (45.2) | NS | AA vs GG vs GA: 0.85  AA vs GA + GG: 0.58  GA + AA vs GG: 0.80 |
| rs749794 | TT: 28 (41.2)  CC: 10 (14.7)  TC: 30 (44.1) | TT: 40 (38.5)  CC: 17 (16.3)  TC: 47 (45.2) | NS | TT vs CC vs TC: 0.92  CC vs TC + TT: 0.77  TC + CC vs TT: 0.72 |
| rs3773640 | AA: 32 (46.4)  TT: 9 (13.0)  AT: 28 (40.6) | AA: 46 (44.2)  TT: 13 (12.5)  AT: 45 (43.3) | NS | AA vs TT vs AT: 0.94  AA vs AT + TT: 0.78  AT + AA vs TT: 0.92 |
| rs3773644 | CC: 42 (60.9)  TT: 5 (7.2)  CT: 22 (31.9) | CC: 51 (49.5)  TT: 5 (4.9)  CT: 47 (45.6) | NS | CC vs TT vs CT: 0.19  CC vs CT + TT: 0.14  CT + CC vs TT: 0.52 |
| rs3773645 | CC: 17 (25.0)  GG: 19 (27.9)  CG: 32 (47.1) | CC: 35 (34.0)  GG: 22 (21.4)  CG: 46 (44.7) | NS | CC vs GG vs CG: 0.39  CC vs CG + GG: 0.21  CG + CC vs GG: 0.32 |
| rs3773652 | AA: 41 (59.4)  GG: 2 (2.9)  AG: 26 (37.7) | AA: 62 (59.6)  GG: 9 (8.7)  AG: 33 (31.7) | NS | AA vs GG vs AG: 0.27  AA vs AG + GG: 0.98  AG + AA vs GG: 0.20 |
| rs2043136 | TT: 55 (79.7)  CC: ---  TC: 14 (20.3) | TT: 72 (69.2)  CC: 2 (1.9)  TC: 30 (28.8) | NS | TT vs CC vs TC: 0.26  CC vs TC + TT: 0.52  TC + CC vs TT: 0.13 |
| rs1346907 | CC: 34 (49.3)  TT: 5 (7.2)  CT: 30 (43.5) | CC: 51 (49.0)  TT: 10 (9.6)  CT: 43 (41.3) | NS | CC vs TT vs CT: 0.85  CC vs CT + TT: 0.98  CT + CC vs TT: 0.59 |
| rs876688 | AA: 25 (36.2)  GG: 8 (11.6)  GA: 36 (52.2) | AA: 37 (35.6)  GG: 17 (16.3)  GA: 50 (48.1) | NS | AA vs GG vs GA: 0.67  AA vs GA + GG: 0.93  GA + AA vs GG: 0.38 |
| rs877572 | GG: 35 (50.7)  CC: 4 (5.8)  CG: 30 (43.5) | GG: 52 (50.5)  CC: 9 (8.7)  CG: 42 (40.8) | NS | GG vs CC vs CG: 0.76  CC vs CG + GG: 0.47  CG + CC vs GG: 0.98 |
| rs9843942 | AA: 29 (42.0)  GG: 10 (14.5)  GA: 30 (43.5) | AA: 29 (28.2)  GG: 27 (26.2)  GA: 47 (45.6) | NS | AA vs GG vs GA: 0.08  AA vs GA + GG: 0.06  GA + AA vs GG: 0.07 |
| rs3773663 | GG: 46 (66.7)  AA: 3 (4.3)  AG: 20 (29.0) | GG: 69 (66.3)  AA: 1 (1.0)  AG: 34 (32.7) | NS | GG vs AA vs AG: 0.39  AA vs AG + GG: 0.30  AG + AA vs GG: 0.97 |
| rs744751 | CC: 53 (76.8)  TT: 1 (1.4)  TC: 15 (21.7) | CC: 83 (80.6)  TT: ---  TC: 20 (19.4) | NS | CC vs TT vs TC: 0.48  CC vs TC + TT: 0.55  TC + CC vs TT: 0.40 |

**Table S3. Norwegian Cohort Subgroup tSNP/SNP Genotype Distributions**

| Gene  SNP | Genotype Counts (%) Cases Delivering < 37.0 weeks gestation | Genotype Counts (%) Cases Delivering ≥ 37.0 weeks gestation | Genotype Counts  (%) Controls | Bivariate Associations  (p value < 0.05) |
| --- | --- | --- | --- | --- |
| *ALK5 (TGFBR1)* |  |  |  |  |
| rs6478974 | AA: 11 (20.0)  TT: 13 (23.6)  TA: 31(56.4) | AA: 5 (22.7)  TT: 3 (13.6)  TA: 14 (63.6) | AA: 23 (36.5)  TT: 13 (20.6)  TA: 27 (42.9) |  |
| rs10739778 | AA: 20 (36.4)  CC: 9 (16.4)  CA: 26 ()47.3 | AA: 13 (59.1)  CC: 1 (4.5)  CA: 8 (36.4) | AA: 28 (44.4)  CC: 5 (7.9)  CA: 30 (47.6) |  |
| rs420549 | GG: 40 (72.7)  CC: 0 (0.0)  GC: 15 (27.3) | GG: 16 (72.7)  CC: 0 (0)  GC: 6 (27.3) | GG: 44 (71.0%)  CC: 2 (3.2%)  GC: 16 (25.8%) |  |
| *ALK1* |  |  |  |  |
| rs3759178 | TT: 21 (38.2)  GG: 7 (12.7)  GT: 27 (49.1) | TT: 8 (36.4)  GG: 1 (4.5)  GT: 13 (59.1) | TT: 24 (38.1)  GG: 3 (4.8)  GT: 36 (57.1) |  |
| rs11169953 | CC: 23 (42.6)  TT: 5 (9.3)  CT: 26 (48.1) | CC: 12 (54.5)  TT: 3 (13.6)  CT: 7 (31.8) | CC: 25 (39.7)  TT: 6 (9.5)  CT: 32 (50.8) |  |
| rs706819 | GG: 34 (61.8)  AA: 4 (7.3)  GA: 17 (30.9) | GG: 9 (40.9)  AA: 2 (9.1)  GA: 11 (50.0) | GG: 29 (46.0)  AA: 2 (3.2)  GA: 32 (50.8) |  |
| *TGFB1* |  |  |  |  |
| rs8179181 | CC: 36 (73.5)  TT: 2 (4.1)  CT: 11 (22.4) | CC: 14 (70.0)  TT: 0 (0)  CT: 6 (30.0) | CC: 37 (62.7)  TT: 6 (10.2)  CT: 16 (27.1) |  |
| rs4803455 | CC: 13 (23.6)  AA: 13 (23.6)  CA: 29 (52.7) | CC: 8 (36.4)  AA: 5 (22.7)  CA: 9 (40.9) | CC: 11 (17.5)  AA: 11 (17.5)  CA: 41 (65.1) |  |
| rs11466314 | GG: 55 (100) | GG: 22 (100) | GG: 63 (100) |  |
| rs1800469 | CC: 30 (54.5)  TT: 5 (9.1)  CT: 20 (36.4) | CC: 9 (40.9)  TT: 5 (22.7)  CT: 8 (36.4) | CC: 34 (54.0)  TT: 4 (6.3)  CT: 25 (39.7) |  |
| rs4803457 | CC: 24 (44.4)  TT: 8 (14.8)  CT: 22 (40.7) | CC: 7 (31.8)  TT: 6 (27.3)  CT: 9 (40.9) | CC: 26 (41.3)  TT: 6 (9.5)  CT: 31 (49.2) |  |
| *ENG* |  |  |  |  |
| rs10987746 | TT: 13 (23.6)  CC: 10 (18.2)  TC: 32 (58.2) | TT: 7 (31.8)  CC: 4 (18.2)  TC: 11 (50.0) | TT: 19 (30.6)  CC: 10 (16.1)  TC: 33 (53.2) |  |
| rs10819309 | GG: 17 (30.9)  AA: 8 (14.5)  GA: 30 (54.5) | GG: 10 (45.5)  AA: 3 (13.6)  GA: 9 (40.9) | GG: 20 (31.7)  AA: 13 (20.6)  GA: 30 (47.6) |  |
| rs10760505 | CC: 23 (41.8)  TT: 7 (12.7)  CT: 25 (45.5) | CC: 7 (31.8)  TT: 3 (13.6)  CT: 12 (54.5) | CC: 25 (39.7)  TT: 9 (14.3)  CT: 29 (46.0) |  |
| rs11792480 | GG: 25 (45.5)  AA: 4 (7.3)  AG: 26 (47.3) | GG: 11 (50.0)  AA: 1 (4.5)  AG: 10 (45.5) | GG: 32 (50.8)  AA: 7 (11.1)  AG: 24 (38.1) |  |
| rs10121110 | AA: 27 (49.1)  GG: 9 (16.4)  AG: 19 (34.5) | AA: 12 (54.5)  GG: 1 (4.5)  AG: 9 (40.9) | AA: 28 (45.9)  GG: 10 (16.4)  AG: 23 (37.7) |  |
| *TGFBR2* |  |  |  |  |
| rs3087465 | GG: 29 (52.7)  AA: 3 (5.5)  AG: 23 (41.8) | GG: 13 (59.1)  AA: 1 (4.5)  AG: 8 (36.4) | GG: 31 (49.2)  AA: 7 (11.1)  AG: 25 (39.7) |  |
| rs6550005 | GG: 28 (57.1)  AA: 2 (4.1)  GA: 19 (38.8) | GG: 13 (59.1)  AA: 0 (0)  GA: 9 (40.9) | GG: 35 (56.5)  AA: 1 (1.6)  GA: 26 (41.9) |  |
| rs11129420 | AA: 5 (9.8)  TT: 11 (21.6)  TA: 35 (68.6) | AA: 7 (31.8)  TT: 5 (22.7)  TA: 10 (45.5) | AA: 18 (29.0)  TT: 8 (12.9)  TA: 36 (58.1) | < 37.0 subgroup  AA vs TT vs TA: 0.03  AA vs TA + TT: 0.02 |
| rs6802220 | GG: 17 (33.3)  AA: 3 (5.9)  AG: 31 (60.8) | GG: 7 (31.8)  AA: 3 (13.6)  AG: 12 (54.5) | GG: 14 (22.6)  AA: 13 (21.0)  AG: 35 (56.5) | < 37.0 subgroup  AA vs AG + GG: 0.03 |
| rs17025785 | TT: 20 (39.2)  CC: 1 (2.0)  TC: 30 (58.8) | TT: 6 (27.3)  CC: 4 (18.2)  TC: 12 (54.5) | TT: 21 (33.9)  CC: 6 (9.7)  TC: 35 (56.5) |  |
| rs4522809 | TT: 12 (23.5)  CC: 15 (29.4)  CT: 24 (47.1) | TT: 6 (27.3)  CC: 3 (13.6)  CT: 13 (59.1) | TT: 14 (22.6)  CC: 9 (14.5)  CT: 39 (62.9) |  |
| rs4955212 | CC: 27 (54.0)  TT: 3 (6.0)  CT: 20 (40.0) | CC: 10 (45.5)  TT: 0 (0)  CT: 12 (54.5) | CC: 41 (66.1)  TT: 2 (3.2)  CT: 19 (30.6) |  |
| rs5020833 | CC: 26 (51.0)  GG: 0 (0)  CG: 25 (49.2) | CC: 11 (50.0)  GG: 3 (13.6)  CG: 8 (36.4) | CC: 25 (40.3)  GG: 4 (6.5)  CG: 33 (53.2) |  |
| rs6809777 | CC: 28 (54.9)  TT: 7 (13.7)  CT: 16 (31.4) | CC: 13 (59.1)  TT: 2 (9.1)  CT: 7 (31.8) | CC: 29 (49.2)  TT: 6 (10.2)  CT: 24 (40.7) |  |
| rs12487185 | AA: 26 (51.0)  GG: 0 (0)  GA: 25 (49.2) | AA: 10 (45.5)  GG: 4 (18.2)  GA: 8 (36.4) | AA: 27 (43.5)  GG: 8 (12.9)  GA: 27 (43.5) | < 37.0 subgroup  AA vs GG vs GA: 0.02  GA + AA vs GG: 0.008 |
| rs11924422 | AA: 12 (23.5)  CC: 6 (11.8)  CA: 33 (64.7) | AA: 6 (27.3)  CC: 3 (13.6)  CA: 13 (59.1) | AA: 23 (37.1)  CC: 8 (12.9)  CA: 31 (50.0) |  |
| rs13083813 | TT: 17 (33.3)  AA: 5 (9.8)  AT: 29 (56.9) | TT: 8 (36.4)  AA: 3 (13.6)  AT: 11 (50.0) | TT: 31 (50.0)  AA: 4 (6.5)  AT: 27 (43.5) |  |
| rs13075948 | CC: 23 (45.1)  TT: 5 (9.8)  CT: 23 (45.1) | CC: 12 (54.5)  TT: 2 (9.1)  CT: 8 (36.4) | CC: 35 (56.5)  TT: 9 (14.5)  CT: 18 (29.0) |  |
| rs1155708 | GG: 21 (41.2)  AA: 1 (2.0)  GA: 29 (56.9) | GG: 11 (50.0)  AA: 4 (18.2)  GA: 7 (31.8) | GG: 20 (32.3)  AA: 11 (17.7)  GA: 31 (50.0) | < 37.0 subgroup  GG vs AA vs GA: 0.02  AA vs GA + GG: 0.01 |
| rs13086588 | TT: 23 (45.1)  GG: 2 (3.9)  GT: 26 (51.0) | TT: 9 (40.9)  GG: 4 (18.2)  GT: 9 (40.9) | TT: 22 (35.5)  GG: 7 (11.3)  GT: 33 (53.2) |  |
| rs2082224 | GG: 34 (66.7)  AA: 3 (5.9)  GA: 14 (27.5) | GG: 11 (50.0)  AA: 0 (0)  GA: 11 (50.0) | GG: 32 (51.6)  AA: 4 (6.5)  GA: 26 (41.9) |  |
| rs1036097 | GG: 10 (19.6)  AA: 11 (21.6)  GA: 30 (58.8) | GG: 9 (40.9)  AA: 5 (22.7)  GA: 8 (36.4) | GG: 20 (32.3)  AA: 8 (12.9)  GA: 34 (54.8) |  |
| rs6792117 | GG: 16 (31.4)  AA: 6 (11.8)  GA: 29 (56.9) | GG: 5 (22.7)  AA: 5 (22.7)  GA: 12 (54.5) | GG: 16 (25.8)  AA: 14 (22.6)  GA: 32 (51.6) |  |
| rs749794 | TT: 26 (47.3)  CC: 1 (1.8)  TC: 28 (50.9) | TT: 9 (40.9)  CC: 3 (13.6)  TC: 10 (45.5) | TT: 25 (39.7)  CC: 7 (11.1)  TC: 31 (49.2) |  |
| rs3773640 | AA: 32 (62.7)  TT: 0 (0)  AT: 19 (37.3) | AA: 10 (45.5)  TT: 1 (4.5)  AT: 11 (50.0) | AA: 33 (54.1)  TT: 6 (9.8)  AT: 22 (36.1) | < 37.0 subgroup  AT + AA vs TT: 0.03 |
| rs3773644 | CC: 15 (29.4)  TT: 10 (19.6)  CT: 26 (51.0) | CC: 8 (36.4)  TT: 1 (4.5)  CT: 13 (59.1) | CC: 15 (24.2)  TT: 7 (11.3)  CT: 40 (64.5) |  |
| rs3773645 | CC: 26 (51.0)  GG: 6 (11.8)  CG: 19 (37.3) | CC: 5 (22.7)  GG: 1 (4.5)  CG: 16 (72.7) | CC: 29 (47.5)  GG: 5 (8.2)  CG: 27 (44.3) |  |
| rs3773652 | AA: 15 (29.4)  GG: 8 (15.7)  AG: 28 (54.9) | AA: 12 (54.5)  GG: 2 (9.1)  AG: 8 (36.4) | AA: 18 (29.0)  GG: 9 (14.5)  AG: 35 (56.5) | ≥ 37.0 subgroup  AA vs AG + GG: 0.04 |
| rs2043136 | TT: 30 (58.8)  CC: 1 (2.0)  TC: 20 (39.2) | TT: 17 (77.3)  CC: 0 (0)  TC: 5 (22.7) | TT: 31 (50.0)  CC: 6 (9.7)  TC: 25 (40.3) | ≥ 37.0 subgroup  TC + CC vs TT: 0.04 |
| rs1346907 | CC: 15 (29.4)  TT: 10 (19.6)  CT: 26 (51.0) | CC: 6 (27.3)  TT: 4 (18.2)  CT: 12 (54.5) | CC: 24 (38.7)  TT: 12 (19.4)  CT: 26 (41.9) |  |
| rs876688 | GG: 20 (39.2)  AA: 8 (15.7)  GA: 23 (45.1) | GG: 7 (31.8)  AA: 5 (22.7)  GA: 10 (45.5) | GG: 27 (43.5)  AA: 8 (12.9)  GA: 27 (43.5) |  |
| rs877572 | GG: 15 (29.4)  CC: 10 (19.6)  CG: 26 (51.0) | GG: 6 (27.3)  CC: 4 (18.2)  CG: 12 (54.5) | GG: 24 (38.7)  CC: 10 (16.1)  CG: 28 (45.2) |  |
| rs9843942 | GG: 17 (33.3)  AA: 9 (17.6)  GA: 25 (49.0) | GG: 4 (18.2)  AA: 4 (18.2)  GA: 14 (63.6) | GG: 25 (40.3)  AA: 12 (19.4)  GA: 25 (40.3) |  |
| rs3773663 | GG: 14 (27.5)  AA: 9 (17.6)  AG: 28 (54.9) | GG: 5 (22.7)  AA: 5 (22.7)  AG: 12 (54.5) | GG: 14 (22.6)  AA: 22 (35.5)  AG: 26 (41.9) | < 37.0 subgroup  AA vs AG + GG: 0.04 |
| rs744751 | CC: 22 (43.1)  TT: 4 (7.8)  TC: 25 (49.0) | CC: 7 (31.8)  TT: 4 (18.2)  TC: 11 (50.0) | CC: 28 (45.2)  TT: 9 (14.5)  TC: 25 (40.3) |  |

**Table S4. Latina Cohort Subgroup tSNP/SNP Genotype Distributions**

| Gene  SNP | Genotype Counts (%) Cases Delivering < 37.0 weeks | Genotype Counts (%) Cases Delivering ≥ 37.0 weeks | Genotype Counts (%) Controls | Bivariate Associations (p value) |
| --- | --- | --- | --- | --- |
| *ALK5 (TGFBR1)* |  |  |  |  |
| rs6478974 | TT: 9 (32.1)  AA: 2 (7.1)  TA: 17 (60.7) | TT: 17 (42.5)  AA: 9 (22.5)  TA: 14 (35.0) | TT: 46 (43.8)  AA: 12 (11.4)  TA: 47 (44.8) |  |
| rs10739778 | AA: 11 (39.3)  CC: 6 (21.4)  CA: 11 (39.3) | AA: 20 (50.0)  CC: 3 (7.5)  CA: 17 (42.5) | AA: 53 (50.5)  CC: 9 (8.6)  CA: 43 (41.0) |  |
| rs420549 | GG: 24 (85.7)  CC: 0 (0)  GC: 4 (14.3) | GG: 32 (80.0)  CC: 0 (0)  GC: 8 (20.0) | GG: 90 (85.7)  CC: ---  GC: 15 (14.3) |  |
| *ALK1* |  |  |  |  |
| rs3759178 | TT: 9 (32.1)  GG: 4 (14.3)  GT: 15 (53.6) | TT: 15 (37.5)  GG: 6 (15.0)  GT: 19 (47.5) | TT: 38 (36.5)  GG: 14 (13.5)  GT: 52 (50.0) |  |
| rs11169953 | CC: 15 (53.6)  TT: 2 (7.1)  CT: 11 (39.3) | CC: 16 (41.0)  TT: 4 (10.3)  CT: 19 (48.7) | CC: 51 (49.5)  TT: 4 (3.9)  CT: 48 (46.6) |  |
| rs706819 | GG: 8 (28.6)  AA: 11 (39.3)  GA: 9 (32.1) | GG: 16 (40.0)  AA: 10 (25.0)  GA: 14 (35.0) | GG: 19 (18.1)  AA: 33 (31.4)  GA: 53 (50.5) | ≥ 37.0 weeks  GG vs AA vs GA: 0.03  GA + AA vs GG: 0.009 |
| *TGFB1* |  |  |  |  |
| rs8179181 | CC: 25 (89.3)  TT: 0 (0)  CT: 3 (10.7) | CC: 31 (81.6)  TT: 1 (2.6)  CT: 6 (15.8) | CC: 84 (84.8)  TT: ---  CT: 15 (15.2) |  |
| rs4803455 | CC: 16 (57.1)  AA: 2 (7.1)  CA: 10 (35.7) | CC: 18 (45.0)  AA: 5 (12.5)  CA: 17 (42.5) | CC: 52 (50.0)  AA: 7 (6.7)  CA: 45 (43.3) |  |
| rs11466314 | GG: 28 (100) | GG: 40 (100) | GG: 105 (100) |  |
| rs1800469 | CC: 7 (25.9)  TT: 7 (25.9)  CT: 13 (48.1) | CC: 8 (20.5)  TT: 10 (25.6)  CT: 21 (53.8) | CC: 25 (24.0)  TT: 23 (22.1)  CT: 56 (53.8) |  |
| rs4803457 | TT: 8 (29.6)  CC: 7 (25.0)  CT: 12 (42.9) | TT: 12 (30.8)  CC: 8 (20.5)  CT: 19 (48.7) | TT: 29 (28.2)  CC: 20 (19.4)  CT: 54 (52.4) |  |
| *ENG* |  |  |  |  |
| rs10987746 | TT: 12 (42.9)  CC: 5 (17.9)  TC: 11 (39.3) | TT: 13 (32.5)  CC: 10 (25.0)  TC: 17 (42.5) | TT: 29 (27.9)  CC: 24 (23.1)  TC: 51 (49.0) |  |
| rs10819309 | GG: 8 (28.6)  AA: 8 (28.6)  GA: 12 (42.9) | GG: 12 (30.8)  AA: 5 (12.8)  GA: 22 (56.4) | GG: 44 (42.3)  AA: 13 (12.5)  GA: 47 (45.2) |  |
| rs10760505 | CC: 12 (42.9)  TT: 3 (10.7)  CT: 13 (46.4) | CC: 14 (35.0)  TT: 9 (22.5)  CT: 17 (42.5) | CC: 37 (35.6)  TT: 19 (18.3)  CT: 48 (46.2) |  |
| rs11792480 | GG: 20 (71.4)  AA: 1 (3.6)  AG: 7 (25.0) | GG: 29 (72.5)  AA: 1 (2.5)  AG: 10 (25.0) | GG: 72 (69.2)  AA: 7 (6.7)  AG: 25 (24.0) |  |
| rs10121110 | AA: 15 (55.6)  GG: 2 (7.4)  AG: 10 (37.0) | AA: 23 (59.0)  GG: 3 (7.7)  AG: 13 (33.3) | AA: 58 (56.3)  GG: 11 (10.7)  AG: 34 (33.0) |  |
| *TGFBR2* |  |  |  |  |
| rs3087465 | GG: 16 (57.1)  AA: 3 (10.7)  AG: 9 (32.1) | GG: 21 (52.5)  AA: 2 (5.0)  AG: 17 (42.5) | GG: 57 (55.3)  AA: 6 (5.8)  AG: 40 (38.8) |  |
| rs6550005 | GG: 21 (72.4)  AA: 1 (3.4)  GA: 7 (24.1) | GG: 31 (77.5)  AA: 0 (0)  GA: 9 (22.5) | GG: 85 (82.5)  AA: 1 (1.0)  GA: 17 (16.5) |  |
| rs11129420 | AA: 12 (41.4)  TT: 6 (20.7)  TA: 11 (37.9) | AA: 11 (27.5)  TT: 8 (20.0)  TA: 21 (52.5) | AA: 38 (36.5)  TT: 13 (12.5)  TA: 53 (51.0) |  |
| rs6802220 | GG: 11 (37.9)  AA: 7 (24.1)  AG: 11 (37.9) | GG: 15 (37.5)  AA: 7 (17.5)  AG: 18 (45.0) | GG: 31 (29.8)  AA: 23 (22.1)  AG: 50 (48.1) |  |
| rs17025785 | TT: 9 (31.0)  CC: 9 (31.0)  TC: 11 (38.0) | TT: 17 (42.5)  CC: 3 (7.5)  TC: 20 (50.0) | TT: 29 (27.9)  CC: 28 (26.9)  TC: 47 (45.2) | ≥ 37.0 subgroup  TC vs CC vs TC: 0.24  CC vs TC + TT: 0.01 |
| rs4522809 | CC: 8 (27.6)  TT: 10 (34.5)  CT: 11 (37.9) | CC: 12 (30.0)  TT: 5 (12.5)  CT: 23 (57.5) | CC: 19 (18.3)  TT: 33 (31.7)  CT: 52 (50.0) | ≥ 37.0 subgroup  CC vs TT vs CT: 0.04  CT + CC vs TT: 0.02 |
| rs4955212 | CC: 16 (55.2)  TT: 5 (17.2)  CT: 8 (27.6) | CC: 9 (23.1)  TT: 5 (12.8)  CT: 25 (64.1) | CC: 45 (44.6)  TT: 11 (10.9)  CT: 45 (44.6) | ≥ 37.0 subgroup  CC vs CT + TT: 0.02 |
| rs5020833 | CC: 12 (41.4)  GG: 7 (24.1)  CG: 10 (34.5) | CC: 20 (50.0)  GG: 5 (12.5)  CG: 15 (37.5) | CC: 41 (39.4)  GG: 21 (20.2)  CG: 42 (40.4) |  |
| rs6809777 | CC: 18 (69.2)  TT: 0 (0)  CT: 8 (30.8) | CC: 29 (72.5)  TT: 1 (2.5)  CT: 10 (25.0) | CC: 68 (65.4)  TT: ---  CT: 36 (34.6) |  |
| rs12487185 | AA: 11 (37.9)  GG: 6 (20.7)  GA: 12 (41.4) | AA: 24 (60.0)  GG: 3 (7.5)  GA: 13 (32.5) | AA: 41 (39.8)  GG: 19 (18.4)  GA: 43 (41.7) | ≥ 37.0 subgroup  AA vs GA + GG: 0.04 |
| rs11924422 | AA: 13 (44.8)  CC: 5 (17.2)  CA: 11 (37.9) | AA: 9 (22.5)  CC: 11 (27.5)  CA: 20 (50.0) | AA: 29 (27.9)  CC: 16 (15.4)  CA: 59 (56.7) |  |
| rs13083813 | TT: 14 (48.3)  AA: 6 (30.7)  AT: 9 (31.0) | TT: 9 (22.5)  AA: 10 (25.0)  AT: 21 (52.5) | TT: 34 (32.7)  AA: 13 (12.5)  AT: 57 (54.8) |  |
| rs13075948 | CC: 19 (65.5)  TT: 0 (0)  CT: 10 (34.5) | CC: 27 (67.5)  TT: 2 (5.0)  CT: 11 (27.5) | CC: 61 (58.7)  TT: 2 (1.9)  CT: 41 (39.4) |  |
| rs1155708 | GG: 8 (27.6)  AA: 7 (24.1)  GA: 14 (48.3) | GG: 21 (52.5)  AA: 4 (10.0)  GA: 15 (37.5) | GG: 45 (43.7)  AA: 16 (15.5)  GA: 42 (40.8) |  |
| rs13086588 | TT: 10 (34.5)  GG: 7 (24.1)  GT: 12 (41.4) | TT: 22 (56.4)  GG: 4 (10.3)  GT: 13 (33.3) | TT: 44 (42.3)  GG: 12 (11.5)  GT: 48 (46.2) |  |
| rs2082224 | GG: 12 (41.4)  AA: 6 (20.7)  GA: 11 (37.9) | GG: 23 (57.5)  AA: 1 (2.5)  GA: 16 (40.0) | GG: 49 (47.6)  AA: 9 (8.7)  GA: 45 (43.7) |  |
| rs1036097 | GG: 11 (37.9)  AA: 6 (20.7)  GA: 12 (41.4) | GG: 7 (17.5)  AA: 14 (35.0)  GA: 19 (47.5) | GG: 27 (26.0)  AA: 23 (22.1)  GA: 54 (51.9) |  |
| rs6792117 | AA: 10 (34.5)  GG: 4 (13.8)  GA: 15 (51.7) | AA: 13 (32.5)  GG: 9 (22.5)  GA: 18 (45.0) | AA: 39 (37.5)  GG: 18 (17.3)  GA: 47 (45.2) |  |
| rs749794 | TT: 10 (35.7)  CC: 6 (21.4)  TC: 12 (42.9) | TT: 18 (45.0)  CC: 4 (10.0)  TC: 18 (45.0) | TT: 40 (38.5)  CC: 17 (16.3)  TC: 47 (45.2) |  |
| rs3773640 | AA: 11 (37.9)  TT: 5 (17.2)  AT: 13 (44.8) | AA: 21 (52.5)  TT: 4 (10.0)  AT: 15 (37.5) | AA: 46 (44.2)  TT: 13 (12.5)  AT: 45 (43.3) |  |
| rs3773644 | CC: 19 (65.5)  TT: 3 (10.3)  CT: 7 (24.1) | CC: 23 (57.5)  TT: 2 (5.0)  CT: 15 (37.5) | CC: 51 (49.5)  TT: 5 (4.9)  CT: 47 (45.6) |  |
| rs3773645 | CC: 8 (27.6)  GG: 12 (41.4)  CG: 9 (31.0) | CC: 9 (23.1)  GG: 7 (17.9)  CG: 23 (59.0) | CC: 35 (34.0)  GG: 22 (21.4)  CG: 46 (44.7) |  |
| rs3773652 | AA: 16 (55.2)  GG: 0 (0)  AG: 13 (44.8) | AA: 25 (62.5)  GG: 2 (5.0)  AG: 13 (32.5) | AA: 62 (59.6)  GG: 9 (8.7)  AG: 33 (31.7) |  |
| rs2043136 | TT: 22 (75.9)  CC: 0 (0)  TC: 7 (24.1) | TT: 33 (82.5)  CC: 0 (0)  TC: 7 (17.5) | TT: 72 (69.2)  CC: 2 (1.9)  TC: 30 (28.8) |  |
| rs1346907 | CC: 15 (51.7)  TT: 0 (0)  CT: 14 (48.3) | CC: 19 (47.5)  TT: 5 (12.5)  CT: 16 (40.0) | CC: 51 (49.0)  TT: 10 (9.6)  CT: 43 (41.3) |  |
| rs876688 | AA: 12 (41.4)  GG: 2 (6.9)  GA: 15 (51.7) | AA: 13 (32.5)  GG: 6 (15.0)  GA: 21 (52.5) | AA: 37 (35.6)  GG: 17 (16.3)  GA: 50 (48.1) |  |
| rs877572 | GG: 16 (55.2)  CC: 0 (0)  CG: 13 (44.8) | GG: 19 (47.5)  CC: 4 (10.0)  CG: 17 (42.5) | GG: 52 (50.5)  CC: 9 (8.7)  CG: 42 (40.8) |  |
| rs9843942 | AA: 12 (41.4)  GG: 3 (10.3)  GA: 14 (48.3) | AA: 17 (42.5)  GG: 7 (17.5)  GA: 16 (40.0) | AA: 29 (28.2)  GG: 27 (26.2)  GA: 47 (45.6) |  |
| rs3773663 | GG: 18 (62.1)  AA: 3 (10.3)  AG: 8 (27.6) | GG: 28 (70.0)  AA: 0 (0)  AG: 12 (30.0) | GG: 69 (66.3)  AA: 1 (1.0)  AG: 34 (32.7) | < 37.0 subgroup  AA vs AG + GG: 0.03 |
| rs744751 | CC: 19 (65.5)  TT: 1 (3.4)  TC: 9 (31.0) | CC: 34 (85.0)  TT: 0 (0)  TC: 6 (15.0) | CC: 83 (80.6)  TT: ---  TC: 20 (19.4) |  |
